# Compliance with Standard Precautions and Its Relationship with Views on Infection Control and Prevention Policy among Healthcare Workers during COVID-19 Pandemic

**DOI:** 10.3390/ijerph18073420

**Published:** 2021-03-25

**Authors:** Eliza Lai-Yi Wong, Kin-Fai Ho, Dong Dong, Annie Wai-Ling Cheung, Peter Sen-Yung Yau, Emily Ying-Yang Chan, Eng-Kiong Yeoh, Wai-Tong Chien, Frank Youhua Chen, Simon Poon, Qingpeng Zhang, Samuel Yeung-Shan Wong

**Affiliations:** 1 Centre for Health Systems and Policy Research, JC School of Public Health and Primary Care, Faculty of Medicine, The Chinese University of Hong Kong, Hong Kong, China; dongdong@cuhk.edu.hk (D.D.); anniewlcheung@cuhk.edu.hk (A.W.-L.C.); b144754@cuhk.edu.hk (P.S.-Y.Y.); yeoh_ek@cuhk.edu.hk (E.-K.Y.); 2 JC School of Public Health and Primary Care, Faculty of Medicine, The Chinese University of Hong Kong, Hong Kong, China; kfho@cuhk.edu.hk (K.-F.H.); emily.chan@cuhk.edu.hk (E.Y.-Y.C.); yeungshanwong@cuhk.edu.hk (S.Y.-S.W.); 3 The Nethersole School of Nursing, Faculty of Medicine, The Chinese University of Hong Kong, Hong Kong, China; wtchien@cuhk.edu.hk; 4 Department of Management Sciences, College of Business, City University of Hong Kong, Hong Kong, China; cbychen@cityu.edu.hk; 5 School of Computer Science, Faculty of Engineering, The University of Sydney, Hong Kong, China; simon.poon@sydney.edu.au; 6 School of Data Science, City University of Hong Kong, Hong Kong, China; qingpeng.zhang@cityu.edu.hk

**Keywords:** workplace infection and prevention guideline, infection control policy, standard precaution, occupational health, nurses, Hong Kong

## Abstract

*Background*: Standard precautions prevent the spread of infections in healthcare settings. Incompliance with infection control guidelines of healthcare workers (HCWs) may increase their risk of exposure to infectious disease, especially under pandemics. The purpose of this study was to assess the level of compliance with the infection prevention and control practices among HCWs in different healthcare settings and its relationship with their views on workplace infection control measures during the COVID-19 pandemic. *Methods*: Nurses in Hong Kong were invited to respond to a cross-sectional online survey, in which their views on workplace infection and prevention policy, compliance with standard precautions and self-reported health during pandemics were collected. *Results*: The respondents were dissatisfied with workplace infection and prevention policy in terms of comprehensiveness (62%), clarity (64%), timeliness (63%), and transparency (60%). For the protective behavior, the respondents did not fully comply with the standard precautions when they were involved in medical care. Their compliance was relatively low when having proper patient handling (54%) and performing invasive procedures (46%). A multivariate analysis model proved that the level of compliance of the standard precautions was positively associated with the satisfaction on infection control and prevention policy among high risk group (0.020; 95% CI: 0.005–0.036), while older respondents had higher level of compliance among the inpatient and outpatient groups (coefficient range: 0.065–0.076). The higher level of compliance was also significantly associated with working in designated team and having chronic condition of the respondents among high-risk and inpatient groups. *Conclusions*: Standard precautions are the most important elements to reduce cross-transmission among HCWs and patients while the satisfaction on infection control and prevention policy would increase the compliance among the high-risk group. An overall suboptimal compliance and poor views on the infection prevention and control guidelines is a warning signal to healthcare system especially during pandemics.

## 1. Introduction

The idea of standard precautions that were developed and practiced long ago in healthcare history is to ensure the minimum infection prevention practices in healthcare. To ensure the adequacy and timely of standard precautions, it was modified and updated in response to different risks of exposure among healthcare workers (HCWs) [1,2,3] in order to improve the well-being of HCWs and patients [4,5]. HCWs, especially nurses, are at risk of acquiring infection through occupational exposure in different healthcare settings than general population in the community [6,7] and the compliance to standard precautions in all situations would be acted as one of the most effective methods to minimum the cross-transmission, regardless of suspected or confirmed infection status of the patients [8]. Despite a significant preparedness and improvement after getting experience from the past epidemics, compliance with optimal practices remains insufficient in general among HCWs and the compliance rates also varied among different elements of the standard precaution [9,10,11].

COVID-19 emerged as a global threat, affecting 94 million people worldwide and causing about 2 million deaths as of January 2021 [12]. As the COVID-19 pandemic progresses, HCWs are the most important resources in providing care for the patients at the frontline in the battle against the disease. However, they are also at higher risk of becoming infected themselves, which could pose a big challenge for epidemic control and lead to the collapse of the healthcare system [13]. A study showed that the prevalence of COVID-19 in HCWs was around 10%, and 29% of infections were due to accident exposure to a patient at a non-COVID-19 facility [14]. Recent evidence also suggests that the risk of asymptomatic spread of COVID-19 to HCWs was presented [15]. Beside the standard precaution suggestions, the WHO has suggested a series of workplace infection control measures at both individual and organization levels for protecting HCWs [16], and strengthening the health systems’ response to COVID-19 [17]. Although workplace infection precaution are important elements to safeguard occupational health in healthcare, it will not do well if individual HCW do not follow them [13]. Thus, implementing agreeable and acceptable workplace infection control guidelines and measures in healthcare settings during an infectious pandemic is necessary to protect HCW’s health and reduce the risk of cross-transmission and infection in the workplace. However, studies on HCW’s views about their workplace infection control policies and measures in healthcare settings are limited [18], and the relationship between the level of compliance during the pandemic is unclear.

To do this, the purpose of the study was to assess level of compliance with infection prevention and control practices among HCWs in different healthcare settings and to explore association between compliance and views on the infection prevention and control practices, and also the characteristics of HCWs. The findings are important to inform strategies and intervention needed to strengthen workplace policy in healthcare settings and sustain the capacity of a healthcare system to combat a pandemic as well as maintain essential health services.

## 2. Materials and Methods

An anonymous cross-sectional survey was conducted among nurses in Hong Kong Special Administrative Region of the People’s Republic of China (HK) through an online platform during COVID-19 pandemic between 26 February and 31 March 2020.

There are 56,723 registered and enrolled nurses in HK based on 2018 statistics [19]. However, only around 32% of the registered nurses and 28% of the enrolled nurses are on active status [20,21]. Registered member records of the Association of Hong Kong Nursing Staff include email contacts for 16,500 nurses; all of them were approached to fill out the online questionnaire. Nurses who were working in any healthcare setting, and either in public or private service provision, were eligible for the survey. Those who did not perform direct patient care were ineligible and excluded from the analysis.

The online self-administered questionnaire was sent to all nurses who had an email addresses in the contacts of the Association of Hong Kong Nursing Staff. A reminder was sent two weeks after the initial email. All participation was voluntary and anonymous. Respondents could choose to withdraw from the study at any point when filling out the questionnaire.

The structured questionnaire was developed based on previous surveys and a review of literature about infectious outbreak in order to explore the protective behavior at workplace regarding compliance with infection control and prevention guidelines and views on workplace infection control and prevention policies. A three-point response scale was used for the measuring the compliance of the infection control and prevention guidelines at workplace (Never = 0, Sometimes = 1 and Always = 2). A total of eight recommended standard precautions at the workplace, which were suggested by WHO [5], were measured, including (1) Hand wash; (2) Using Personal Protective Equipment (PPE) to protect from exposure to infectious materials; (3) Adopting respiratory hygiene/cough etiquette principles; (4) Proper patient handling (cleaning and disinfecting); (5) Environmental cleaning and disinfection; (6) Proper handling of textiles and laundry; (7) Performing invasive procedure (injection, lumber puncture, e.g.,); (8) Handling of needles and other sharp objects. Regarding views on infection control and prevention policy in the workplace, in terms of comprehensiveness, clarity, timeliness, and transparency, a five-point Likert-type scale was adopted for the response scale (very dissatisfied to very satisfied). Information about the demographics (including age, gender, employment status, marital status, had/had not children, and chronic condition), and working characteristics of the nurses (including service type—high risk, inpatient or outpatient, service setting—public or private hospital/clinic and whether worked in designated team to care for the COVID-19 patients) were also collected.

Data management and analysis were conducted using R version 4.0.3 (R Foundation for Statistical Computing, Vienna, Austria). Any *p* < 0.05 was regarded as statistically significant. Descriptive information of the adoption of standard precaution and the views on the infection control and prevention policy at workplace were reported. In order to indicate the level of the compliance for each respondent, an average precaution score was calculated among the eight measured standard precautions if they were applied. For example, if the nurse answered “sometimes—score 1” in question 1–4, answered “Always—score 2” in question 5–6, answered “No—score 0” in question 7 and reported “N.A.—not counted in the summative score” in question 8, then her average precaution score is equal to (1 + 1 + 1 + 1 + 2 + 2 + 0)/7 = 1.14. For the views on the infection control and prevention policy at workplace, a summative policy score was suggested if a good model fit of the factors structure among the measured perception views on the infection control and prevention policy in terms of comprehensiveness, clarity, timeliness, and transparency in the confirmatory factor analyses (CFA). Goodness of fit indices such as the comparative fit index (CFI), goodness of fit (GFI), adjusted goodness of fit (AGFI), the Tucker–Lewis index (TLI) and normed fit index (NFI) were used to assess the adequacy of model fit. According to the previous criteria, CFI, TLI and AGFI greater than 0.9, GFI and NFI greater than 0.95 represent a good model fit [22]. Internal consistency and reliability of the policy score was examined by Cronbach’s alpha coefficient and the corrected item-total correlation. It is acceptable if Cronbach’s alpha might be greater than 0.70. The corrected item-total correlation is the correlation between each item and the sum of the other items in a scale. Each item in a scale should contribute to measuring a core common construct of the scale, otherwise it may be excluded. Corrected inter-item correlations using the criterion of 0.3 or higher were used to identify items related to the full scale [23,24]. Chi-square/fisher exact tests were used to indicate the categorical responses of the adoption of precaution and the views on the infection policy among the different service setting while Kruskal–Wallis or Pairwise Wilcoxon Rank Sum tests were applied for the average precaution score and the summative policy scores. The Tobit regression was used to explore the associations between the views on infection control and prevention policy in the workplace and the level of precaution adoption among the three different groups of service setting. Significant predictors at *p*-value < 0.2 in the univariate analyses were included in the multivariate regression analysis.

## 3. Results

### 3.1. Background Characteristics

A total of 839 nurses responded to the survey during COVID-19 pandemic. Of them, 33 reported that they worked in academic, school, or office setting; therefore, only 806 valid responses representing front-line nurses in health care setting were retained in the analysis. Of the 806 respondents, about half of them were aged under 40 (53%) and most of them were female (88%), married (59%), without child (58%), and without chronic disease (78%). For the employment status, a majority worked in the public service provision (76%) and only 15% of them were involved in the designated team to take care the COVID-19 patients. The working fields were grouped into three types: high-risk group including those worked in accidental and emergency department, intensive care unit, infectious ward, isolation ward and operation room (25%), inpatient (43%) and outpatients (32%).

### 3.2. Views on Workplace Infection Control and Prevention Policy

More than half of respondents were dissatisfied with the policy in terms of comprehensiveness (62%), clarity (64%), timeliness (63%), and transparency (60%). In the CFA, all of the goodness of fit indices were fulfilled in the proposed summative policy score (comparative fit index (CFI) = 0.996, Tucker-Lewis index (TLI) = 0.987, goodness of fit (GFI) = 0.990, normed fit index (NFI) = 0.995, adjusted goodness of fit (AGFI) = 0.948)]. The factor loadings of the measured perception views ranged from 0.867 to 0.932 and were significant at *p* < 0.05. In addition, the Cronbach’s α is 0.948 and the corrected item-total correlation ranged from 0.843 to 0.894, which indicated the good internal consistency reliability of the summative policy score for further analysis. Thus, the summative policy was estimated and the findings indicated that the score was 11.2 (SD = 4.5) out of 20. The responses were different among the three working groups and a particularly higher proportion of dissatisfaction was found in those from the inpatient group and reported significantly lower level of satisfaction score on the workplace infection control and prevention policy than those in outpatient setting (*p* < 0.05) (Table 1).

### 3.3. Adoption of Standard Precaution at Workplace

Regarding the protective behavior in the workplace, the respondents did not have a full level of compliance with the standard precaution when they involved in the medical care. Most of them expressed that they always adopted hand wash (99%), environmental cleaning and disinfection (86%), respiratory hygiene/cough etiquette principles (80%) and proper handling of textiles and laundry (80%). However, the compliance was lower when handling of needles and other sharp objects (74%) and using PPE to protect from exposure to infectious materials (73%). Nearly half of them had not always adopted standard precaution when performing invasive procedure (46%) and having proper patient handling (54%). The mean precaution score was 1.7 (SD = 0.3). The level of compliance “having proper patient handling”, “adopting respiratory hygiene/cough etiquette principles”, “performing invasive procedure (e.g., injection, lumber puncture)” and “using PPE to protect from exposure to infectious materials” were significantly varied among different service groups. According to the statistical analysis, high-risk group scored higher than inpatient group while inpatient group scored higher than outpatient group (*p* < 0.05) (Table 2).

### 3.4. Factors Associated with the Compliance of Standard Precaution

The results of the univariate regressions to explore the relationship between the level of precaution compliance, the satisfaction on infection control and prevention policy and other demographic characteristics for each service group are reported in Table 3. The satisfaction of the workplace infection control and prevention policy was positively associated with the level of compliance for standard precaution in high risk and inpatient groups. Age was also significantly associated with higher level of compliance in all service groups. Moreover, of the married or cohabited respondents, those had children and chronic conditions particularly had had higher level of compliance among high risk group while working in the designated team were additionally associated with the level of compliance in inpatient group (Table 3). In the multivariate analysis (Table 4), the satisfaction of the infection control and prevention policy score was still positively associated to have higher level of compliance of the standard precaution among high risk group but not in inpatient and outpatient groups. On the other hand, older respondents significantly had higher level of compliance among the inpatient and outpatient groups only. For those “working in designated team” and “having chronic condition” tended to have higher level of compliance among inpatient group and high-risk group, respectively.

## 4. Discussion

To our knowledge, this is the first study to explore whether the infection control and prevention guidelines at workplace facilitated the compliance among nurses during the COVID-19 pandemic. Regarding to the protective behaviors of the nurses, the level of compliance was not the optimal and varied among each of the specific measured components of infection prevention and control guidelines. A relatively higher proportion of HCWs were found to by always compliant with hand wash, environmental cleaning and disinfection, adopting respiratory hygiene/cough etiquette principles and having proper handling of textile and laundry. Nevertheless, the nurses had poor performance, especially when performing invasive procedure and having proper patient handling. A relatively similar finding has been observed in hand hygiene compliance among HCWs in another local study during pandemics [25]. The findings were also consistent with previous studies that the compliance of infection prevention and control guidelines was suboptimal [11,26,27,28]. Our study identified areas to improve nurses’ knowledge of high-risk procedure related to performing invasive procedure and proper patient handling during second wave of COVID-19 pandemic.

In the study, the model further suggested a significant positive relationship between adoption of standard precaution, views on policy and age among high-risk group and inpatient/outpatient group, respectively. The level of satisfaction of infection control policy was positively associated with compliance of standard precaution for the nurse in high-risk group. It seems that the nurse would have higher level of compliance with infection prevention and control guideline if they feel more satisfied on workplace infection control policy [29], which will, in turn, preserve healthcare workers’ and patients’ well-being. However, for the nurses in lower risk working setting—inpatient and outpatient groups—there is no significant relationship with satisfaction of policy in compliance of standard precaution. Younger age was positively associated with lower compliance with infection control guideline in inpatient and outpatient group. It may imply that younger nurses (especially not in designated team) may have less experience and training to fully compliance with infection control guideline. In addition, those did not work in designated team tended to have lower level of compliance among the inpatient group may due to the high burden of patient flows during the pandemic. Thus, nurses working in all settings may benefit from additional training on the importance and implementation of infection control guideline that could minimize the infection risk.

In addition, the findings suggested that the current workplace policy was perceived to unable to facilitate nurse’s protective behaviors in the workplace especially among the nurse in high-risk group. This trend was also observed in outpatient and inpatient settings. In order to increase the work engagement in the infection control and prevention, the organization should provide a regular infection control training among all HCWs from different healthcare setting in understanding their challenges of the implantation of infection control guideline. If these issues are not addressed, it may create a healthcare capacity crisis in coming days [30].

The study results could serve to guide the management level in developing interventions to create supportive work environments for the health and safety of nurses. With the experience of SARS, MERS and Swine Flu, the infrastructure of healthcare system, infection control policy and training in HK are strengthened [31,32]; however, our study found that HCW’s satisfaction with infection control and prevention policy in the workplace was not as high as expected. There were high proportion of discrepancy views on the workplace infection and prevention policy in terms of comprehensiveness, clarity, timeliness, and transparency. The proportion of dissatisfaction was significantly higher among the nurse in high-risk setting. The nurses in high-risk setting were more eager for more policy support. It might reflect their inadequate coping behavior as the infection control procedures were frequently modified because of the evolving understanding of COVID-19 pandemic. The findings urged to health organization implementing comprehensive and feasible workplace infection control guidelines and measures in healthcare settings during an infectious pandemic is necessary to protect HCW’s health and reduce the risk of cross-transmission and infection in the workplace. Besides the suggested standard precaution, the organization should also explore and implement other considerable precautions with training or workshop to support HCWs when handling the high-risk procedures for the medical care during pandemics [33,34]

There was no study that directly considered the impact of stress or mental health problems on adoption of standard precaution according to our literature search. However, some studies considered lack of access to up-to-date information and communication is a sources of healthcare personnel anxiety related to the COVID-19 epidemic [35]. It might be worth conducting some mediation model to explore whether stress or mental health is a mediator variable between adoption of standard precaution and views on policy in further analysis.

## 5. Limitation

In the study, the responses were lower than expected. It may be due to the fatigue of nurses during the pandemics as they are stretched to handle the increasing patient load due to COVID-19 and, thus, only a few them were willing to respond the survey. Selection bias may arise due to the low response rate, which affected the prevalence of the compliance with the standard precaution and the satisfaction level of the infection control and prevention policy. However, the impact of bias would be minimized when examining the relationship between the standard precaution and the satisfaction of infection control and prevention policy in a multivariate model [36]. On the other hand, the design of cross-sectional survey can explore the prevalence of the target outcome and the association between the exposure and outcome simultaneously, but it may not derive the causality relationships from the collected data. Thus, there is a temporal relationship between exposure and outcome variables concluded. Further qualitative studies such as in-depth interview or focus group discussion are suggested to examining the possible causal relationship between exposure and outcome.

Another limitation was a lack of validity in the absence of face-to-face interviews. However, anonymity may allow HCWs to feel more comfortable to express their genuine views towards the workplace policy and health outcome, it prevents the tracing and investigation of responders. Despite of these limitations, our study provides an important insight into existing shortcomings in the infectious control policy and measure in healthcare setting for international reference to address HCW’s need and concern regarding to the occupational safety and health.

## 6. Conclusions

Standard precaution or infection control and prevention guideline are the most important elements to reduce cross-transmission among healthcare workers and patients. An overall suboptimal compliance and poor views on the infection prevention and control guidelines is a warning signal to healthcare system especially during pandemics. Immediate improvement actions to strengthen infection control and prevention policy and training for younger nurses are needed. Healthcare workers everywhere are putting themselves on the frontlines battling the disease in pandemics. Their dissatisfaction and inadequate support from their workplace will seriously affect their well-being and may create a healthcare capacity crisis in coming days. Thus, supporting workplace policy plays an important role in the protection of healthcare worker. The organization should create new strategies and intervention to strengthen workplace policy in healthcare settings and to increase sustainability of healthcare system for handling new challenges from the pandemic and ongoing needs of health system users.

## Figures and Tables

**Table 1:** Views on workplace infection control and prevention policy by service setting.

Policy	Total	High Risk	Inpatient	Outpatient	*p*-Value
**Comprehensiveness**					0.010 *
Very dissatisfied	136 (16.9)	24 (11.9)	70 (20.3)	42 (16.2)	
Dissatisfied	218 (27.0)	65 (32.2)	93 (27.0)	60 (23.1)	
Not sure	149 (18.5)	41 (20.3)	68 (19.8)	40 (15.4)	
Satisfied	263 (32.6)	65(32.2)	100 (29.1)	98 (37.7)	
Very satisfied	40 (5.0)	7 (3.5)	13 (3.8)	20 (7.7)	
**Clarity**					<0.001 *
Very dissatisfied	146 (18.1)	26 (12.9)	74 (21.5)	46 (17.7)	
Dissatisfied	231 (28.7)	76 (37.6)	107 (31.1)	48 (18.5)	
Not sure	135 (16.7)	30 (14.9)	60 (17.4)	45 (17.3)	
Satisfied	265 (32.9)	64 (31.7)	93 (27.0)	108 (41.5)	
Very satisfied	29 (3.6)	6 (3.0)	10 (2.9)	13 (5.0)	
**Timely**					0.004 *
Very dissatisfied	138 (17.1)	22 (10.9)	74 (21.5)	42 (16.2)	
Dissatisfied	216 (26.8)	67 (33.2)	91 (26.5)	58 (22.3)	
Not sure	153 (19.0)	39 (19.3)	69 (20.1)	45 (17.3)	
Satisfied	261 (32.4)	68 (33.7)	96 (27.9)	97 (37.3)	
Very satisfied	38 (4.7)	6 (3.0)	14 (4.1)	18 (6.9)	
**Transparency**					0.044 *
Very dissatisfied	147 (18.2)	31 (15.3)	73 (21.2)	43 (16.5)	
Dissatisfied	197 (24.4)	62 (30.7)	84 (24.4)	51 (19.6)	
Not sure	142 (17.6)	35 (17.3)	64 (18.6)	43 (16.5)	
Satisfied	276 (34.2)	65 (32.2)	108 (31.4)	103 (39.6)	
Very satisfied	44 (5.5)	9 (4.5)	15 (4.4)	20 (7.7)	
**Policy Score (mean + SD)**	11.2 + 4.5	11.2 + 4.155	10.7 + 4.4	11.9 + 4.7	0.001 *

* *p*-value < 0.05 are bold.

**Table 2:** Level of compliance of standard precautions at workplace by service setting.

Variable	Total	High Risk	Inpatient	Outpatient	*p*-Value
**Hand wash**					0.465
Never	1 (0.1)	0 (0.0)	1 (0.3)	0 (0.0)	
Sometime	9 (1.1)	1 (0.5)	3 (0.9)	5 (1.9)	
Always	793 (98.8)	199 (99.5)	339 (98.8)	255 (98.1)	
**Having proper patient handling**					<0.001 *
Never	74 (9.5)	5 (2.5)	25 (7.4)	44 (18.2)	
Sometime	347 (44.6)	60 (30.2)	166 (49.3)	121 (50.0)	
Always	357 (45.9)	134 (67.3)	146 (43.3)	77 (31.8)	
**Adopting respiratory hygiene/cough etiquette principles**					0.037 *
Never	27 (3.5)	2 (1)	10 (3)	15 (6)	
Sometime	127 (16.3)	29 (14.8)	61 (18.3)	37 (14.9)	
Always	624 (80.2)	165 (84.2)	263 (78.7)	196 (79)	
**Environmental cleaning and disinfection**					0.240
Never	8 (1)	0 (0.0)	4 (1.2)	4 (1.6)	
Sometime	101 (12.8)	21 (10.4)	50 (14.7)	30 (12.1)	
Always	680 (86.2)	180 (89.6)	286 (84.1)	214 (86.3)	
**Proper handling of textiles and laundry**					0.082
Never	13 (1.7)	6 (3)	3 (0.9)	4 (1.6)	
Sometime	145 (18.7)	31 (15.7)	74 (22.4)	40 (16)	
Always	619 (79.7)	160 (81.2)	253 (76.7)	206 (82.4)	
**Handling of needles and other sharp objects**					0.498
Never	25 (3.3)	4 (2)	10 (3)	11 (4.7)	
Sometime	171 (22.5)	46 (23.4)	78 (23.6)	47 (20.1)	
Always	565 (74.2)	147 (74.6)	242 (73.3)	176 (75.2)	
**Performing invasive procedure (eg injection, lumber puncture)**					<0.001 *
Never	104 (15)	15 (7.9)	34 (11)	55 (28.4)	
Sometime	212 (30.6)	47 (24.7)	104 (33.7)	61 (31.4)	
Always	377 (54.4)	128 (67.4)	171 (55.3)	78 (40.2)	
**Using PPE to protect from exposure to infectious materials**					<0.001 *
Never	42 (5.5)	0 (0.0)	15 (4.5)	27 (11.5)	
Sometime	162 (21.1)	33 (16.7)	57 (17.1)	72 (30.8)	
Always	562 (73.4)	165 (83.3)	262 (78.4)	135 (57.7)	
**Precaution score (mean + SD)**	1.7 + 0.3	1.8 + 0.3	1.7 + 0.3	1.6 + 0.3	<0.001 *

* *p*-value < 0.05 are bold. PPE―Personal Protective Equipment.

**Table 3:** Univariate analysis on the level of compliance of standard precautions.

Variable	Total	High Risk		Inpatient		Outpatient	
	*N* (%)	Coef (95% CI)	*p*-Value	Coef (95% CI)	*p*-Value	Coef (95% CI)	*p*-Value
**Age**		0.087 (0.029, 0.145)	0.003 *	0.076 (0.039, 0.112)	<0.001 *	0.065 (0.011, 0.119)	0.019 *
18–29	174 (21.6)						
30–39	251 (31.1)						
40–49	218 (27)						
≥50	163 (20.2)						
**Gender**							
F	705 (87.5)	ref	-	ref	-	ref	-
M	101 (12.5)	0.014 (−0.158, 0.186)	0.872	0.072 (−0.044, 0.187)	0.220	0.118 (−0.069, 0.304)	0.215
**Employment status**							
Full time	765 (94.9)	ref	-	ref	-	ref	-
Part time	41 (5.1)	0.27 (−0.302, 0.841)	0.355	0.196 (−0.009, 0.401)	0.060	0.076 (−0.11, 0.261)	0.424
**Service type**							
Public	611 (75.8)	ref	-	ref	-	ref	-
Private/NGO	195 (24.2)	0.008 (−0.161, 0.177)	0.926	−0.009 (−0.117, 0.099)	0.870	0.002 (−0.104, 0.108)	0.976
**Worked in designated team**							
No	683 (84.7)	ref	-	ref	-	ref	-
yes	123 (15.3)	−0.021 (−0.145, 0.104)	0.744	0.221 (0.04, 0.402)	0.020 *	0.098 (−0.156, 0.352)	0.449
**Marital status**							
Single/Widow/ Separation	328 (40.7)	ref	-	ref	**-**	ref	-
Married/ Cohabited	478 (59.3)	0.166 (0.043, 0.289)	0.008 *	0.114 (0.036, 0.191)	<0.001 *	0.041 (−0.07, 0.153)	0.469
**Had children**							
No	468 (58.1)	ref	-	ref	**-**	ref	-
Yes	338 (41.9)	0.246 (0.115, 0.376)	<0.001 *	0.089 (0.009, 0.168)	0.030 *	−0.021 (−0.125, 0.084)	0.696
**Living status**							
Alone	52 (6.5)	ref	-	ref	-	ref	-
Family/friends/ other	754 (93.5)	0.193 (−0.021, 0.407)	0.077	−0.079 (−0.238, 0.08)	0.330	0.181 (−0.055, 0.417)	0.132
**Had chronic conditions**							
No	626 (77.7)	ref	-	ref	-	ref	-
yes	180 (22.3)	0.254 (0.083, 0.425)	0.004 *	0.053 (−0.044, 0.15)	0.290	0.08 (−0.036, 0.196)	0.178
**Policy score (mean + SD)**	1.699 + 0.307	0.023 (0.008, 0.038)	0.003 *	0.009 (0.001, 0.018)	0.040 *	0.008 (−0.003, 0.019)	0.167

* *p*-value < 0.05 are bold. NGO—Non-governmental organization.

**Table 4:** Multivariate analysis on the level of compliance of standard precautions.

Variable (Coef (95% CI))	High Risk Group		Inpatient Group		Outpatient Group	
	Estimate	*p*-Value	Estimate	*p*-Value	Estimate	*p*-Value
**Age**	0.008 (−0.06, 0.075)	0.823	0.058 (0.015, 0.1)	0.008 *	0.056 (0, 0.113)	0.048 *
**Employment status**						
Full time	-	-	ref	-	-	-
Part time	-	-	0.143 (−0.059, 0.345)	0.164	-	-
**Worked in designated team**						
No	-	-	ref	-	-	-
yes	-	-	0.233 (0.055, 0.411)	0.010 *	-	-
**Marital status**						
Single/Widow/Separation	ref	-	ref	-	-	-
Married/Cohabited	0.031 (−0.126, 0.187)	0.703	0.067 (−0.05, 0.184)	0.263	-	-
**Had children**						
No	ref	-	ref	-	ref	-
Yes	0.175 (0.002, 0.348)	0.047	−0.024 (−0.144, 0.096)	0.693	0.049 (−0.07, 0.168)	0.421
**Had chronic conditions**						
No	ref	-	-	-	-	-
yes	0.210 (0.038, 0.382)	0.017 *	-	-	-	-
**Policy score (mean + SD)**	0.020 (0.005, 0.036)	0.009 *	0.004 (−0.005, 0.013)	0.380	0.007 (−0.004, 0.018)	0.206

* *p*-value < 0.05 are bold.

## Data Availability

The data presented in this study are available on request from the corresponding author. The data are not publicly available due to privacy and ethical reasons.
